# Application of Biosensors Based on Lipid Membranes for the Rapid Detection of Toxins

**DOI:** 10.3390/bios8030061

**Published:** 2018-06-26

**Authors:** Georgia-Paraskevi Nikoleli, Dimitrios P. Nikolelis, Christina G. Siontorou, Stephanos Karapetis, Marianna-Thalia Nikolelis

**Affiliations:** 1 Laboratory of Inorganic & Analytical Chemistry, School of Chemical Engineering, Department 1, Chemical Sciences, National Technical University of Athens, 9 Iroon Polytechniou St., 15780 Athens, Greece; dnikolel@chem.uoa.gr (G.-P.N.); stevekara@chem.uoa.gr (S.K.); 2 Laboratory of Environmental Chemistry, Department of Chemistry, University of Athens, Panepistimiopolis-Kouponia, 15771 Athens, Greece; 3 Laboratory of Simulation of Industrial Processes, Department of Industrial Management and Technology, School of Maritime and Industry, University of Piraeus, 18534 Pireus, Greece; csiontor@unipi.gr (C.G.S.); m.nikolelis@gmail.com (M.-T.N.)

**Keywords:** biosensors, lipid membranes, toxins

## Abstract

Lipid assemblies in the form of two dimensional films have been used extensively as biosensing platforms. These films exhibit certain similarities with cell membranes, thus providing a suitable means for the immobilization of proteinaceous moieties and, further, a number of intrinsic signal amplification mechanisms. Their implementation in the detection of toxins yielded reliable and fast detectors for in field analyses of environmental and clinical samples. Some examples are presented herein, including aflatoxin and cholera toxin detection. The conditions and parameters that determine the analytical specifications of the lipid membrane sensors are discussed, advantages and technology bottlenecks are reviewed, and possible further developments are highlighted.

## 1. Introduction

Biosensors exploit the interplay between two components in order to provide useful information about a target compound: A biological moiety and a physicochemical transducer. The interaction between the target compound and the biological moiety (receptor) provides a biochemical signal that is readily converted into an electric signal by the transducer (electrochemical, optical, piezoelectric, calorimetric, etc.).

Although sophisticated techniques such as liquid chromatography (LC) provide accurate results, biosensor devices offer a much higher throughput of samples at a lower cost and with less training of personnel. In addition, biosensor devices are not bulky and can be used for in the field measurements. The uses of biosensors are extremely varied, with food and environmental analysis as an emerging and growing application.

Nanomaterial-based biosensors represent the integration of material science, molecular engineering, chemistry and biotechnology. Nanomaterials can greatly improve the sensitivity and specificity of biomolecule detection and have great potential to constructing devices for molecular recognition, food and environment monitoring, clinical analysis and pathogen diagnosis.

**Figure 1:**
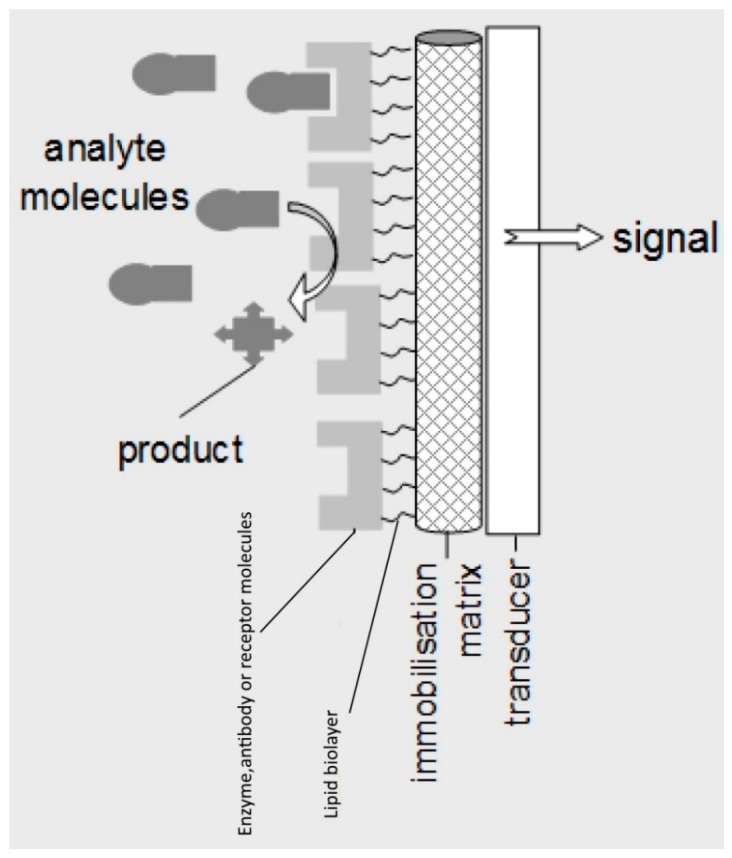
Schematic of a lipid membrane-based biosensor.

Lipid membrane offers a nature-like environment for the immobilization and control of the receptor. At the same time, the physics of the film per se offer a means for signal transduction and amplification: The biochemical interaction affects significantly lipid–protein and lipid–lipid interactions, thus disrupting the continuity of the assembly leading to detectable alterations of transmembrane ion permeability. Figure 1 provides a schematic of a lipid membrane-based biosensor.

### 1.1. Mechanism of Signal Generation in Lipid Based Biosensors

The signal generation mechanism using lipid membrane based biosensors depends on the type of fabrication of the lipid membrane and whether the membrane is freely-suspended, filter- or metal-supported or supported on a polymer.

The mechanism of signal generation for freely-suspended or filter supported BLMs has been provided in the literature [1,2,3]. In the case of lipid membrane based biosensors that use an enzyme as a transduction/recognition element, the enzyme is located in the lipid film in such a way that the hydrophobic portions of the enzyme is incorporated into the lipid, leaving the active site of the enzyme at the aqueous interface [1,4]. A detailed comparison of the profiles and magnitudes of the current transients that were obtained in our previous works [1,5] using flow injection technique for acetylcholine determination indicate a number of important similarities and substantial differences. The most significant similarity is the appearance of well-defined transient currents in the same direction in both experiment as the hydronium ion concentration at the BLM surface is altered during the enzymatic reactions. The quantitative signal of these BLMs biosensors was primarily based on diffusion effects that controlled the time delay of the appearance of the current transients.

The transients of the flow injection analysis experiments [2] have a maximum magnitude of about 50 pA and duration of about 10 s. The mechanism of signal generation was investigated using differential scanning calorimetric studies (DSC) with egg PC/DPPA vesicles [2]. These studies have shown that the phase transition temperature for the gel to liquid-crystalline phase of these vesicles was pH depended. The results indicated that at pH 8.0 and at 25 °C, a gel and liquid-crystalline phase co-exists and this is induced by the presence of Ca^2+^. This phase separation results in an increase of the number of defects in the phase structure of a lipid film and therefore the ion permeation through these membranes increases. Each BLM surface is in equilibrium with its adjacent bulk solution and any alterations of either of the membrane surfaces will result in a reorganization of the BLM double layer. In this case there are two possible mechanisms of signal generation: The appearance of structural defects (i.e., artificial ion-channel gating) will drive the ions to diffuse through the defect sites. The number of ions that passes through the BLMs depends on the speed of both of “channel” opening and the diffusion of ions from the surface of the lipid membrane to the bulk solution. In the case that there is a slow “channel” formation, this will result that the majority of ions will be dissipated into the bulk solution; in the case that the “channel” opening is fast, this will result in the passage of an appreciable portion of the ions through the lipid film. The former phenomena were noticed when diffusion controlled changes of the pH at one side of a BLM (hydrolytic enzyme reactions) took place with concurrent observations of a charging current transients [1,5] while the latter phenomena were observed in the flow experiments in which the defects number varies dynamically as the pH of the carrier electrolyte solution alters; these results indicate how amplification of analytical signal of bilayer lipid membrane based transducers may be made [2].

The mechanism of signal generation using metal supported lipid membranes (s-BLMs) has been reported in the literature [6,7]. Previous studies have shown a model of a potential across metal supported (s-BLMs) and evaluated the structure of the inner lipid layer (facing the Ag wire electrode). It has been reported that the lipid head groups bind to the electrode by interactions of oxygen atoms of the phosphate groups of the lipids with silver ions in the metal lattice [6,7]. Furthermore, chloride ions can diffuse through the lipid membrane during the initial BLM process of stabilization. Chloride ions would interact with the Ag metal to form AgCl. The fast response of a s-BLMs that contain gramicidin to ammonium ions provides the conclusion that these BLMs have a bimolecular structure. No discernable threshold transients were noticed during these experiments; this suggests that critical capacitive events due to changes of surface potential of BLMs were absent [5]. Chloride ions of the electrolyte solution are not the dominant factor that controls ion balance at the metal surface and transport through ion channels. Chloride ions from the AgCl may form an electrostatical bond with cations to reduce charge in a hydrophobic matrix, and concentration gradients result in a diffusion of such undissociated salts through membranes into bulk solution. However, there are also other examples reported in the literature that would include: The possibility that structural defects between phase domains of a lipid membrane could provide transient water containing pathways capable of salt transport, hexagonal or micellar phase structure which act as carriers of salts, or lipid flip-flop across BLMs where the lipid head group would interact with a salt.

The signal generation mechanism of polymer lipid membranes was explored by Raman and IR spectrophotometry and DSC experiments. The results of Raman spectrophotometry have exhibited that there was a shift of the peak at 1176 cm^−1^ to 1195 cm^−1^, which shows that there is a strong electrostatic interaction between the carboxylate groups of methylacrylate and the –NR_3_^+^ groups of phosphatidylcholine (PC) [8,9]. When a “receptor” (which can be enzyme, antibody, or natural or artificial receptor) is added to the polymerization mixture, then this peak was shifted back to 1177 cm^−1^. These results suggest that the “receptor” destabilizes the bonding between the –COO^−^ groups of methylacrylate and the –NR_3_^+^ groups of PC. IR spectrophotometry was utilized to explore the mechanism of signal generation. The IR spectrum of the polymer has shown two characteristic bands: the band at 1729 cm^−1^ which corresponds to the carbonyl stretching of the carboxylic groups of methacrylate and a peak at 1631 cm^−1^ which corresponds to the carboxylate groups due to a self dissociation [10,11,12]. When the lipid molecules were incorporated within the polymer structure, the peak at 1631 cm^−1^ was strongly increased, which suggests an ionization of the carboxylic groups and an electrostatic interaction between the groups of methylacrylate and the –NR_3_^+^ groups of PC. However, when the “receptor” was incorporated within polymer, a decrease of the peak at 1631 cm^−1^ and a raise of the peak at 1729 cm^−1^ were noticed. These results have shown that there is a strong electrostatic interaction between the carboxylate groups of methylacrylate and the –NR_3_^+^ groups of PC and that the “receptor” destabilizes these bonds.

When the enzyme is incorporated in the structure of polymerized lipid membrane, the polymer with the lipid film becomes more flexible due to the incorporation of the enzyme in the structure of the polymer [13] The incorporation of a “receptor” changes the phase structure of the lipid to more fluid [13]. The mechanism of signal generation was studied using DSC technique. The phase transition temperature (*T*_m_) of dipalmiloyl phosphatidic choline (DPPC) in our experiments using an enzyme incorporated in the polymer lipid film was found to be 73.9 ± 1.0 °C; note that the *T*_m_ of DPPC liposomes without any “receptor” is 42 °C [11]. However, presently DPPC in lipid films is polymerized and exists in the solid state. When the enzyme was incorporated within the lipid film, the phase transition temperature was decreased to 57.2 ± 1.0 °C. These results exhibit that the fluidity of the lipid membrane is increased and therefore the number of defects is expected to be increased. In the presence of urea, the phase transition of the lipid film is increased to 61 ± 1 °C, These results exhibit that the lipid membrane becomes more rigid.

**Figure 2:**
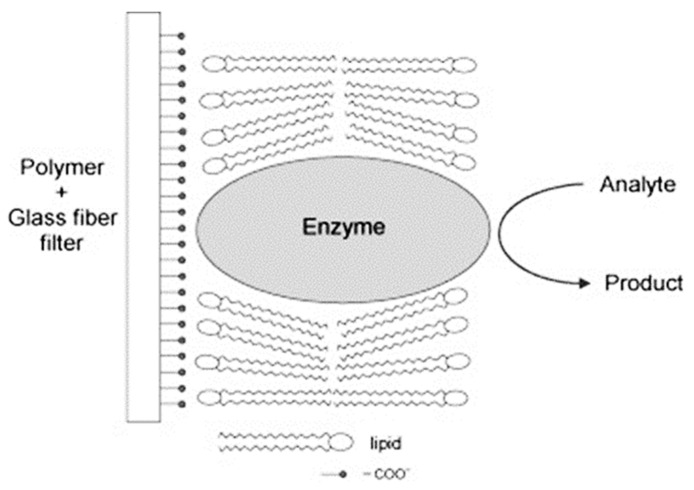
Schematic diagram of the lipid film-enzyme biosensor (with permission from ref. [14]).

The above results exhibit that when the lipid is on a polymer, there is an electrostatic interaction between the –COO^−^ groups of methylacrylate and the –NR_3_^+^ groups of phosphatidylcholine; these electrostatic interactions make the polymer more rigid. As a result the polymethacrylate molecules with the incorporated lipid membrane cannot vibrate or turn around its *x*-axis. The incorporation of an enzyme destabilizes these electrostatic bonds and therefore the polymer can now vibrate or turn around its *x*-axis and the structure becomes more fluid. In the presence of the urea, the film becomes more rigid again. In this case, the lipid membrane structure alters from liquid crystalline to gel. A schematic diagram of the lipid film-enzyme sensor showing its structure is presented in Figure 2.

**Figure 3:**
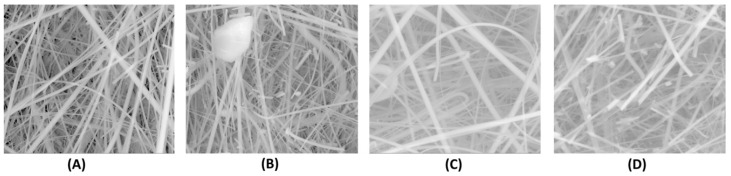
SEM images of (**A**) polymer without the lipid, (**B**) polymer with the lipid, (**C**) polymer with the lipid after the incorporation of Anti-STX, and (**D**) same as (**C**) but a drop of toxin is placed on the filter. Magnification is 2000× (nm). (With permission from ref. [15]).

In order to further investigate the mechanism of signal generation, electron scanning microscopy (SEM) experiments were performed in the presence and absence of a toxin (i.e., anti-STX) [15]. The SEM images of the polymer without the lipid (Figure 3A) exhibit that the structure is not crystalline and different as that in the presence of the lipid (Figure 3B); In this case, the structure becomes crystalline. SEM images of the structure of the polymer with the lipid after the incorporation of the toxin (Figure 3C) shows that the presence of the toxin, the structure becomes again similar to that of the non-crystalline image of Figure 3A. The structure becomes again crystalline after when a drop of the toxin is deposited onto the filter (Figure 3D). These results are in agreement with previous obtained in the literature [16]; saxitoxin has shown the ability to provide electrostatic interactions with lipid membranes [17], this could affect the fluidity and phase structure of the lipid film [18]. These changes in the phase structure of the lipid film, alters the surface potential and electrostatics of the lipid membrane and these phenomena effect the fluidity of the membrane.

### 1.2. State of Art of Lipid Based Biosensors

Lipid membranes represent an appropriate biocompatible structure for the development of new types of biosensors with fast response (on the order of a few seconds) and high sensitivity (i.e., nanomolar detection limits) that may eventually be used in health diagnosis and in field applications for food analysis and environmental monitoring. Most of these biosensors are cost efficient, easy-to-use, fast responding and are good alternative to mostly expensive, time consuming standard analytical and screening methods (i.e., chromatographic procedures or mass spectroscopy). These devices will be able to be either regenerated for uses multiple times or be used as disposable sensors in a single format.

Recent advances in lipid membrane based biosensors have provided devices that exhibit long shelf life and adequate operational stability for environmental and food analysis. The state of art and advantages of lipid film based biosensors are fast response times, nM detection limits, high sensitivity and selectivity, the biosensing surface can be easily and reproducibly prepared and renewed. These biosensors can be easily packed into a portable in the field device for the rapid quality preliminary assessment for life-threatening contaminants and bioterrorism attacks.

These sensors reveal detection limits in the nanomolar range. The most important aspect of the present efforts is to provide a commercial portable sensor that can be used for in-field and market applications by non skilled personnel. Present technology starts to offer chemo- or biosensors based on lipid film technology that can be portable and be used by non-specialized personnel for rapid in situ detections of various compounds.

## 2. Toxins

A toxin is a poisonous substance that is produced within living cells or organisms, created usually by artificial processes. The term was first used by organic chemist Ludwig Brieger (1849–1919) [19]. Toxins can be small molecules, peptides, or proteins that cause diseases on contact with or absorption by body tissues they interact with biological macromolecules such as enzymes or cellular receptors. Toxins vary greatly in their toxicity, which ranges from minor (such as a bee sting) to deadly (such as botulinum toxin).

Toxins are often distinguished from other chemical agents by their method of production. It simply means it is a biologically produced poison. There was an ongoing terminological dispute between NATO and the Warsaw Pact over whether to call a toxin a biological or chemical agent, in which the NATO opted for biological agent, and the Warsaw Pact, like most other countries in the world, for chemical agent.

According to an International Committee of the Red Cross review of the Biological Weapons Convention, “Toxins are poisonous products of organisms; unlike biological agents, they are inanimate and not capable of reproducing themselves”, and “Since the signing of the Constitution, there have been no disputes among the parties regarding the definition of biological agents or toxins” [20].

Toxins can also be classified as either exotoxins, being excreted by an organism, or endotoxins, that are released mainly when bacteria are lysed. An exotoxin is a toxin that is secreted by bacteria and causes damage to the host by destroying cells or disrupting normal cellular metabolism [21]. They are highly dangerous and cause major damage to the host. Gram negative pathogens secrete vesicles in the out space of membranes that contain lipopolysaccharide endotoxin. As a result, they contribute to the eukaryote processes of membrane vesicle trafficking, which is quite active at the host-pathogen interface.

Some well-known exotoxins are: Botulinum toxin produced by Clostridium botulinum; Corynebacterium diphtheriae toxin, produced during diphtheria; tetanospasmin produced by Clostridium tetani. The toxic properties of most exotoxins can be inactivated by heat or chemical treatment to produce a toxoid. The latter retain their antigenic specificity and can be used to produce antitoxins and, in the case of diphtheria and tetanus toxoids, are used as vaccines.

The term “biotoxin” is sometimes used to explicitly confirm the biological origin of these toxins. Biotoxins maybe classified to fungal biotoxins, microbial biotoxins, plant biotoxins, or animal biotoxins. Toxins produced by microorganisms are important virulence determinants responsible for microbial pathogenicity and/or evasion of the host immune response [22].

Biotoxins are highly complex. For example, the venom of the cone snail that contains dozens of small proteins, each of them targets a specific nerve channel or receptor. Biotoxins in nature have the function of Predation, such as in the spider, snake, scorpion, jellyfish, and wasp and of Defense as in the bee, ant, termite, honey bee, wasp, and poison dart frog.

1. Some of the more well-known types of biotoxins include:
2. Cyanotoxins, produced by cyanobacteria.
3. Dinotoxins, produced by dinoflagellates.
4. Necrotoxins cause necrosis in the cells and destroy all types of tissue.
5. Neurotoxins primarily affect the nervous systems of animals. They mostly consist of ion channel toxins that disrupt ion channel conductance.
6. Myotoxins are small, basic peptides found in snake and lizard venoms, they cause muscle tissue damage by a non-enzymatic receptor-based mechanism.
7. Cytotoxins are toxic at the level of individual cells. Typical examples of these toxins are:

- Ricin, from castor beans
- Apitoxin, from honey bees
- T-2 mycotoxin, from certain toxic mushrooms

Aflatoxins are produced by certain fungi that occur in a wide range of foods. Aflatoxin B_1_, is a recognized carcinogen. When aflatoxin B_1_ is ingested by cows, it is secreted as its hydroxylated metabolite, aflatoxin M_1_ (AFM_1_). Due to the carcinogenicity of AFM_1_, the determination of this toxic compound in milk is of increasing interest [23]. An action level of 0.5 ppb (i.e., 1.5 nM) for AFM_1_ in milk has been established by the U.S. Food and Drug Administration; however, this level may be legislated lower in some European countries (i.e., 0.15 nM) [24].

Cholera toxin (CT) is a bacterial polypeptide and is included in a group of biologically active compounds which interact with specific gangliosides in natural and artificial membranes. It is now well known that the interaction between cholera toxin and the ganglioside GM1 causes the activation of adenylate cylase [25], a phenomenon considered to be a classical example for signal transduction through biological membranes [26]. Cholera toxin has been shown to form channels in artificial bilayers containing GM1 [27]. Binding of toxin to gangliosides results in the generation of macromolecular and surface complexes [28,29]. CT is a protein enterotoxin secreted by the bacterium *Vibrio cholerae* that can cause an epidemic disease and lead to rapid dehydration, acidosis, and death within a few h. Most of the cholera cases are reported in underdeveloped countries, and it is estimated that CT causes ca. 120,000 deaths per year. CT has become a very important compound in the weapons against bioterrorism, and there has been increasing interest to developing of rapid, selective, sensitive and portable biosensors for its determination [29,30].

AFB1is produced mainly by molds, e.g., *Aspergillus flavus* and *Aspergillus parasiticus*. It was described in early 1960s as potent human carcinogen (first hazard class in accordance with the classification of the International Agency for Research on Cancer) [31].

Saxitoxin (STX) is the best-known paralytic shellfish toxin (PST) and has both potent marine biotoxin and neurotoxin action. Ingestion of STX, usually by consumption of shellfish contaminated by toxic algal blooms, is responsible for the life threatening PSP. An amount as low as 1 mg dose of the toxin from a single serving of 400 g of contaminated shellfish meat (2.5 ppm STX) is fatal to humans [32]. The European Food and Safety Authority set the acute reference dose to 0.5 μg STX per kg body weight [32], while most of the countries have established the drinking water standard to 1–3 μg/L of STX [33].

## 3. Lipid Membranes

The lipid bilayer (or phospholipid bilayer) is a thin polar membrane made of two layers of lipid molecules. These membranes are flat sheets that form a continuous barrier around all cells. The cell membranes of almost all living organisms and many viruses are made of a lipid bilayer, as are the membranes surrounding the cell nucleus and other sub-cellular structures. The lipid bilayer is the barrier that keeps ions, proteins and other molecules where they are needed and prevents them from diffusing into areas where they should not be. Lipid bilayers are ideally suited to this role, even though they are only a few nanometers in width [34], they are impermeable to most water-soluble (hydrophilic) molecules. Bilayers are particularly impermeable to ions; this allows cells to regulate salt concentrations and pH by transporting ions across their membranes using proteins called ion pumps.

Biological bilayers are usually composed of amphiphilic phospholipids that have a hydrophilic phosphate head group and a hydrophobic tail that consists of two fatty acid chains. Phosphatidylcholine is the major lipid constituent of lipid membranes. Other typical phospholipids are phosphatidyl acid, phosphatidylethanolamine, phosphatidylserine, phosphatidylinositol, etc. The latter lipids can alter the surface chemistry of a bilayer membrane and can serve as signal transporter to the cells [35]. The head groups alter both the surface structure and the charge of the lipid membrane. The bilayer membrane can either be in a solid gel phase state at lower temperatures or in a fluid (liquid crystalline) state at higher temperatures. The fluidity of the lipid film depends on the acyl chain of the phospholipid and the temperature that the lipid becomes fluid is called transition temperature. The physical and chemical properties of the membrane such as resistance, mechanical properties, etc. depend on both the acyl chain and the head group of the phospholipid. Many of these properties have been studied with the use of artificial “model” bilayers produced in a lab. Vesicles made by model bilayers have also been used clinically to deliver drugs.

Biological membranes typically include several types of molecules other than phospholipids. A particularly important example in animal cells is cholesterol, which helps influences the phase structure of the bilayer and decrease its permeability. Cholesterol also helps regulate the activity of certain integral membrane proteins. Integral membrane proteins function when incorporated into a lipid bilayer, and they are held tightly to lipid bilayer with the help of an annular lipid shell. Because bilayers define the boundaries of the cell and its compartments, these membrane proteins are involved in many intra- and inter-cellular signaling processes. Certain kinds of membrane proteins are involved in the process of fusing two bilayers together. This fusion allows the joining of two distinct structures as in the fertilization of an egg by sperm or the entry of a virus into a cell. Lipid bilayers are fragile and are prone to mechanical and electrical noise. Experiments on bilayers often require physicochemical advanced techniques like scanning electron microscopy, atomic force microscopy, differential scanning calorimetry, etc.

## 4. Biosensors Based on Lipid Membranes

Since the discovery of model bilayer lipid membranes (BLM) by Mueller et al. [36], there have been attempts to use them in biosensing applications. However, these free-standing BLMs were fragile and not suitable for long-term use. Important step toward practical applications of lipid films took place in 1980 when Thompson et al. [37] introduced supported bilayer lipid membranes (sBLM) formed on a polyamide polymer substrate. sBLMs can be formed on various substrates, such as metals, polymers, microfiltration membranes, carbon nanotubes, graphene, porous silicon, etc. They can be modified by proteins or artificial receptors, enzymes, antibodies, channel formers and carriers that serve as signal transducers or receptors for detection of analyte. In contrast with BLM, sBLM are more stable and in certain cases can be stored at low temperature (4 °C) for several days without loss of stability and functionality. In addition, many physical techniques can be applied for study the properties of sBLMs [38]. Recent advances in stabilization of lipid bilayer have resulted in preparation of sBLM based biosensors for detection of a variety of analytes. However, the large disadvantage of sBLMs was that they could not be reused and served as single shot sensors.

Stabilization of lipid film biosensors was first accomplished by Nikolelis group [39] by using glass fiber filters to support and stabilize the lipid membrane. However, these membranes were not stable outside an electrolyte solution in the air. Later on, lipid polymer membranes supported on glass fiber were shown to be stable in air [40]. Recently, the deposition of these polymer lipid films on graphene electrode [41] has shown to offer advantages such as increased sensitivity and selectivity, decreased response times and provided biosensors for remote sensing. 

Lipid membranes represent an appropriate biocompatible structure for the development of new types of biosensors with fast response (on the order of a few seconds) and high sensitivity (i.e., nanomolar detection limits) that may be used in health diagnosis and in field applications for food analysis and environmental monitoring. These biosensors are cost efficient, easy-to-use and are good alternative to expensive and time consuming standard analytical methods (i.e., chromatography or mass spectroscopy).

### 4.1. Stabilized Free Standing BLMs

Stabilization of solventless bilayer lipid membranes (BLMs), and the use of stabilized BLMS as flow detectors was reported in a paper by Nikolelis et al. [42]. Microporous filters composed of glass fibers, polytetrafluoroethylene (PTFE) and polycarbonate (nominal pore sizes from 1 to 5 μM) served as interfaces to separate two solution compartments and the micropores in the filter media acted as supports for formation and stabilization of BLMs. Optimization of the flow cell design, and the chemical composition and methods for preparation of stabilized BLMs, are described therein [42]. Lipid membranes were composed of mixtures of phosphatidyl choline and phosphatidic acid and responded rapidly to pH changes of the carrier electrolyte solution. Signals reproducibly appeared within a few s that followed the injection of an electrolyte of different pH than the carrier. Signals were in the form of a single ion current transient with magnitude of tens of pA and a duration of seconds. The mechanism of signal generation was explored by physicochemical techniques such as differential scanning calorimetry, etc. The results show that a phase transition within a lipid membrane can be triggered by pH alterations of the electrolyte solution. These lipid membranes were stable for flow through experiments, however, were not stable in the air.

### 4.2. Polymer Lipid Membranes

**Figure 4:**
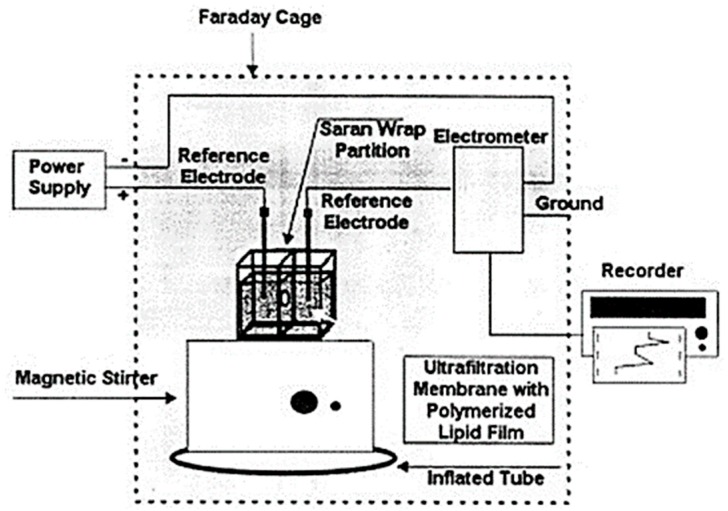
Simplified version of the experimental set-up used. The microporous ultrafiltration membrane (diameter of approximately 9 mm) with the polymerized lipid film was placed between the two plastic layers of the Saran-Wrap, with the filter centered on a 0.32 mm orifice. The Saran-Wrap is clamped between two Perspex blocks and extends their limits, so no leakage of the electrolyte solution will occur around the edges of the blocks. Stirring can be made with a magnetic flea in a small well. (Reprinted from ref. [40] with permission).

The design, preparation and storage of polymer lipid membranes on microporous filtering media such as glass fibers was provided in one of our previous papers [40]. The paper has investigated in terms of simplicity for membrane preparation and for stability of BLMs after storage in air when are immersed in electrolyte solution Briefly their preparation technique is as follows, 5 mg of phosphatidyl choline (PC) was mixed with 0.070 mL of methacrylic acid, 0.8 mL of ethylene glycol dimethacrylate, 8 mg of 2,2′-azobis-(2-methylpropionitrile) and 1.0 mL of acetonitrile. Methacrylic acid was the functional monomer for the polymerization, ethylene glycol dimethacrylate was the cross-linker and 2,2′-azobis-(2-methylpropionitrile) (AIBN) was the initiator. The mixture was sparged with nitrogen for about 1 min and sonicated for 30 min. This mixture could be stored in the refrigerator. For the preparation of the stabilized polymer lipid films, 0.15 mL of this mixture was spread on the microfilter and was left at 60 °C for 12 h. This microporous filter disk (diameter of approximately 9 mm) with the stabilized lipid film was placed between the two plastic layers, with the filter centered on the 0.32 mm orifice (Figure 4). The Saran-Wrap partition with the filter in place was then clamped between the Plexiglas chambers. The partition extended beyond the limits of the edges of the chambers so that no ion current leakage could occur around partition. The presence of the lipid membrane was verified by the magnitude of the ion current obtained. When the ion current stabilized (over a period of less than 5 min), the solution of the analyte is injected in one solution compartment using continuous gentle stirring. These stabilized lipid films provide similar response to freely suspended BLMs (i.e., artificial ion gating events in the form of transient signals) and can function for repetitive uses after storing in air.

Stabilized polymeric lipid membranes can be excellent host matrices for maintenance and transduction of the activity for the number of biochemically selective species such as enzymes, antibodies and receptors [40]. Significant progress has recently been achieved in stabilization and analytical applications of biosensors based on lipid films. Filter-supported bilayer lipid membranes within the polymer could be stored in air for more than a month and provided responses to various analytes [43,44,45]. In one of our recent papers, the polymerization process took place by using UV irradiation instead of heating the polymeric mixture at 60 °C [46].

In this recent paper [46], the polymerization process took place by using UV irradiation instead of heating the lipid mixture at 60 °C. This process retains the activity of an enzyme, whereas heating may deactivate it. The technique also of preparation of stabilized lipid films by polymerization by heating at 60 °C was rather empirical and the mechanism was never studied. In this report [46], the method for the preparation of stabilized lipid membranes was studied using Raman spectroscopy. The results have indicated that the kinetics of polymerization is completed within 4 h and the mechanism of polymerization was investigated by using Raman spectroscopy and differential scanning calorimetry [9,46].

This has made possible the practical use of techniques based on lipid films for chemical sensing because it allows incorporation of natural ion-channels in these lipid films and will allow commercialization of these devices.

This process retained the activity of an enzyme (i.e., acetylcholinesterase) [46], whereas heating may deactivate it. One of the most important criteria to assess the stability and possibility for the practical applications of lipid-based biosensors is the capability of the storage at ambient room temperature conditions. The analytical stability, reproducibility and reusability of such devices have been shown in several published research articles [46,47,48].

### 4.3. Polymer Lipid Membranes on Graphene Electrode

The technique for the construction of biosensors based on polymer lipid membranes on graphene electrode was reported recently [41]. Briefly, the graphene electrode has been prepared with a homogeneous dispersion (ca. 0.4 mg/mL) in N-methyl-pyrrolidone (NMP) through mild sonication (using a Bandelin SONOREX Digital 10P sonicator, Sigma-Aldrich, Taufkirchen, Germany) for 180 h and centrifugation at 700 rpm for 2 h [49]. It has been noticed that this increased sonication time is required because the size of the flakes is severely reduced, which is a critical parameter for several applications.

This graphene suspension has been poured onto a copper wire (d = 0.25 mm) mounted on a glass fiber filter; the evaporation of the organic solvent was carried out using a fan heater. This copper wire has been utilized to establish the connection for the extraction of voltage signals for the calibration curve.

Stabilized lipid films were prepared by polymerization, as previously described in literature [46,47,48]. The polymerization could take place either by using UV irradiation or thermal polymerization; 5 mg of DPPC were added to a mixture of 0.070 mL of methylacrylic acid, 0.8 mL of ethylene glycol dimethacrylate, 8 mg of 2,2-azobis-(2-methylpropionitrile), 1.0 mL of acetonitrile. The mixture was spurged with nitrogen for about 1 min and sonicated for 30 min. For the preparation of the stabilized lipid films, 0.15 mL of this mixture was spread on the microfilter. The filter with the mixture was then irradiated using the UV deuterium lamp. Polymerization was completed within 4 h. Alternatively, the polymerization could take place by thermal polymerization in 80 °C, but the time of polymerization is longer. These membranes were stable to store in air for periods of more than two months.

The enzyme, antibody or receptor was incorporated in bilayer lipid membranes (BLMs) during polymerization by spreading 10 mL of the “receptor” suspension along with the polymerization mixture (i.e., for the preparation of the GM1-incorporated lipid films, the microfilter was spread with 0.15 mL of the polymerization mixture and 10 mL of receptor suspension). These electrodes can be used once (and then disposed of) or repetitively (after regenerated); when not in use, they are kept at 4 °C.

**Figure 5:**
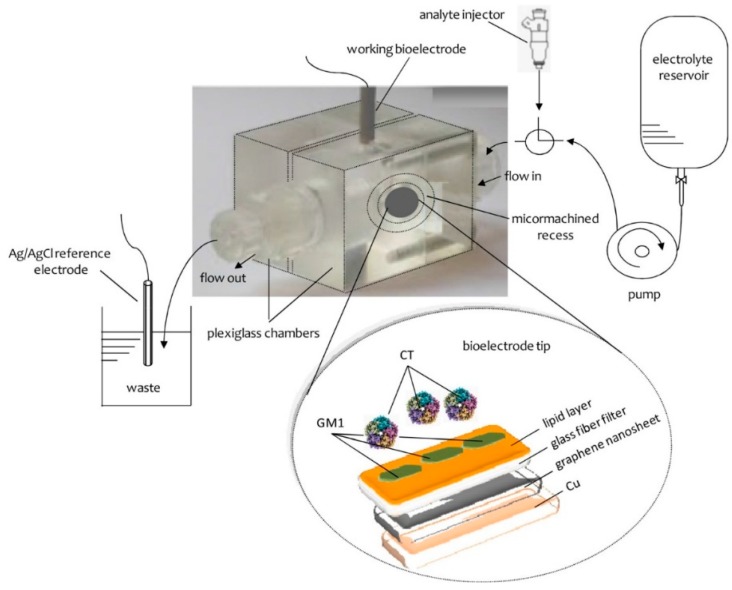
Schematic of the experimental set-up and the bioelectrode edge surface. (Reprinted from ref. [50]).

The preparation of the potentiometric biosensor concluded after the encapsulation of the filter-supported polymerized lipid film onto the copper wire containing graphene nanosheets (Figure 5). The bioelectrode can be stored at 4 °C when not in use; it remains stable for over three months.

## 5. Applications on Biosensors Based on Lipid Membranes for the Detection of Toxins

The interactions of aflatoxin MI with bilayer lipid membranes (BLMs) composed from egg phosphatidylcholine (PC) were investigated [51]. The interactions of aflatoxin M1 (AFM1) with these membranes were found to be electrochemically transduced by BLMs in the form of a transient current signal with duration of seconds and appeared within 7 s after exposure of the membranes to this toxin. BLMs composed of PC have provided maximized sensitivity. The mechanism of signal generation was explored. The mechanism is due to alterations of the membrane surface electrostatics (i.e., reorganization of the electrochemical double layer of membranes) which results in a charging current signal) due to the adsorption of AFM1. The magnitude of the transient current signal was related to the concentration of AFM1in bulk solution in the concentration range 2–15 nM. The application of the electrochemical transduction system for the determination of aflatoxin MI in skimmed milk was explored.

The rapid, sensitive and selective electrochemical flow injection monitoring of AFM1 using stabilized filter-supported BLMs was reported in the literature [52]. Injections of AFM1 were made into the flowing streams of a carrier electrolyte solution (i.e., KCl), and a transient current signal with duration of seconds reproducibly appeared less than 10 s after exposure of the lipid membranes to the toxin. The magnitude of this signal was related linearly to the concentration of AFM1, with detection limits subnanomolar. The mechanism of signal generation was explored by physicochemical techniques (i.e., differential scanning calorimetric) experiments and the results have shown phase structure alterations of the lipid membranes.

To further explore the mechanism of signal generation of these electrochemical results [51], DSC experiments that simulate the interactions of these freely suspended BLMs with AFM_1_ were performed. Vesicles composed of pure egg PC were not examined, as this lipid is known to have a phase transition temperature, *T*_m_, of −10 °C, and the fluidity of BLMs composed of egg PC is not significantly altered above this temperature [53].

The *T*_m_ of vesicles composed of 35% DPPA (pH 8.0 and in the presence of 1.0 mM Ca^2+^) was found to be 23.6 °C and did not practically change in the presence of the toxin, these results indicate that there is no adsorption of AFM1 onto the surface of the lipid membranes. When vesicles which were composed of 10% DPPA at pH 7.0 or 15% DPPA at pH 3.5 (in the absence of Ca^2+^) were examined, there was a shift in *T*_m_. In the former case, the melting temperature was 53.9 °C in the absence of AFM_1_ and decreased to 47.9 °C in the presence of 0.305 μM AFM_1_ and further to 45.4 °C when using 1.22 μM AFM_1_. In the latter, the transition temperature, *T*_m_, decreased from 57.9 to 54.4 °C in the presence of 0.305 μM AFM_1_ and further to 42.6 °C for 1.22 μM AFM_1_. These alterations of the transition temperature may be attributed to the fact that AFM_1_ destabilizes the phase structure of the lipid membranes by forming hydrogen bonds with the lipid molecules and, therefore, disrupting their bonding network and the lipid−lipid continuation [54].

The technique was applied for the rapid flow injection determination of AFM1 in milk and milk preparations. The effect of potent interferences, such as proteins and lipids, was investigated, and the results have shown that the interferences from these milk constituents can be eliminated by modulation of the flow rate of the carrier solution so that not to allow adsorption of lipids and proteins in the BLMs surface. AFM1 could be determined in continuous flowing systems with a rate of 4 samples min^−1^. Repetitive cycles of injection of AFM1 showed no signal degradation during each cycle.

The effect of cholera toxin on the capacitance and conductance of lipid monolayers that contained different gangliosides has been reported in the literature [55]. Cholera toxin (at a concentration of 0.2 μg/mL) increased the dielectric capacitance of monolayers containing 1% and 3% GM1 from 1.5 μF/cm^2^ up to 6 μF/cm^2^ and their ionic permeability by two orders of magnitude. No significant effect of cholera toxin was observed on monolayers containing other gangliosides, e.g., GT1b. Agitation of the suspension or imposing a *cis*-positive diffusion potential facilitates the penetration of the CT-GM1 surface complexes into the bilayer subsequently enhancing the release of T1^+^. A model consisting of CT-GM1 complexes forming high dielectric, ion-permeable perturbations in the monolayers or in the *cis*-half of bilayers is suggested. Incorporation of the complex to form a channel through the bilayer is induced by a *cis*-positive electric field or by mechanical and interfacial stresses on the bilayer.

The development of a one-shot electrochemical sensor for AFM_1_ has been also reported [56]. The interactions of AFM1 with these Langmuir-Blodgett films were found to be electrochemically transduced as a transient current signal with a duration of s and appeared within 7 s after exposure of the membranes to AFM1. The magnitude of the transient current signal was related to the concentration of AFM1 in the range 2–15 nM. The present preliminary work included novel applications of BLM-based films in real samples and explored the matrix effects in the electrochemical transduction for the determination of AFM1 in skimmed milk.

The interactions of AFM1 with self-assembled metal-supported bilayer lipid membranes and its effects on DNA hybridization have been investigated [57]. Alterations of electrochemical signals due to DNA hybridization were used for rapid detection of this toxin. The interactions of AFM1 with these membranes (composed of egg PC) have provided ion current increases which reproducibly appeared within ca. 8–10 s after the lipid membranes was exposed to the toxin. The magnitudes of the current signals were related to the toxin concentration, which could be determined within the range of 1.9–20.9 nM. Further on AFM1 was found to affect the kinetics and time of signal generation due to DNA hybridization, which was electrochemically monitored by using s-BLMs. The “receptor” oligomer was single stranded deoxyribonucleic acid (ssDNA) thymidylic acid icosanucleotide that terminated with a C 16 alkyl chain to assist incorporation into s-BLMs (dT20 -C16). The target oligomer was deoxyadenylic acid icosanucleotide (dA20). The “receptor” was incorporated into s-BLMs and complementary dA 20 (cDNA) was injected into the stirred bulk electrolyte solution. The electrochemical ion current across s-BLMs was found to increase due to the presence of ssDNA and decrease due to the formation of double stranded DNA (dsDNA). The toxin reduced the initial rate of signal alterations and increased the time of equilibrium. This provided a means for the rapid (less than 1 min) and sensitive (detection limit 0.5 nM) detection of AFM1 based on measurements of the initial rate of hybridization.

A multi-assay flow through sensor has been proposed for the detection of AFM1 in cheese [58]. Stabilized filter-supported (BLMs) were used as detectors. Single stranded dT20-C16 was incorporated into the membranes. The incorporation of dT20-C16 in BLMs lowered the detection limit for the detection of this toxin by one to four orders of magnitude as compared with the detection limit obtained in the absence of DNA. This biosensor provides the possibility to continuously monitor this toxin at concentrations that approached those that could be of interest as set by the U.S. Food and Drug Administration and most European countries. Injections of AFM1 were made into flowing streams of a 0.1 M KC1 electrolyte solution, and a transient current signal with duration of s appeared 12 s after exposure of the BLM to the toxin. The magnitude of this signal was related linearly to the concentration of the toxin with detection limits at sub-nM concentration levels. The effect of interferents, such as proteins and lipids, was explored. The interferences from proteins could be eliminated by adjustment of the {low rate of the carrier electrolyte solution. The technique was applied for the rapid flow injection determination of aflatoxin M1 in cheese samples with a rate of at least 4 samples min^−1^. Repetitive cycles of injection of AFM1 have shown no signal degradation during each cycle for experiments that attempted over 30 cycles of detection.

Lipid films were deposited on the sensing surface in an optical biosensor instrument [59]. The membranes were mixtures of biologically occurring lipids. Eight surfaces were prepared, some of which contained various glycolipids as minor components. One was supplemented with membrane proteins. The binding of six protein toxins (cholera toxin, cholera toxin B subunit, diphtheria toxin, ricin, ricin B subunit, staphylococcal enterotoxin B) and of bovine serum albumin at pH 7.4 and pH 5.2 to each of the sensor surfaces was investigated. Each of the seven proteins gave a distinct binding pattern. The assay is rapid and simple, with no need for reagents. The lipid sensor surface is readily regenerated after binding and very stable. The concept with mixed lipid layers and assays at different pH values gives numerous combinations and could be applicable for developing a sensor for protein toxins.

The electrochemical interactions of cholera toxin with ganglioside GM1 stabilized on lipid films have been, also, investigated [60]. The toxin was injected into the flowing streams of a carrier electrolyte solution, the flow of the solution stops for 5 min and an ion current transient was recorded. The signal magnitude was correlated to the concentration of the toxin with detection limits of 0.06 μM. The method was applied in blood serum samples.

A paper that reports the preparation of a device that is based on a polymer stabilized lipid film with incorporated a natural receptor that can be used for the rapid electrochemical detection of cholera toxin in blood serum and potentially could be commercialized has appeared in the literature [60]. The method has rapid response times [order of min] and detection limits at micromolar levels. The biosensing surface could be renewed easily and high reproducibility. The lipid film-based interface at the biosensing surface is providing a biocompatible environment that resists to nonspecific adsorption of serum constituents, thus providing a low background signal in the assay. The results have shown that this device could permit sensitive, selective and reusable detection of CT with highly resistance to nonspecific adsorption. The advantages of the developed biosensor over immunoassay techniques in CT detection has also been described in this report [59]. The receptor was incorporated into the supported polymer lipid film sensing surface and was constructed through a simple polymerization step avoiding the activity loss. Secondly, the receptor binds rapidly to CT with strong affinity and specificity that permits rapid detection. The implementation of lipid-based detection probe allowed efficient incorporation of GM1. It was expected that this strategy might furnish an ideal protocol for sensitive detection of various protein targets in clinic diagnosis and medical research. This technique has similar advantages to high performance liquid chromatographic methods and could be used as a complimentary method for the rapid detection of this toxin as a bioterrorism weapon.

A novel piezoelectric biosensor has been reported in the literature for the rapid detection of cholera toxin [61]. This device was based on a lipid film that contained ganglioside (GM1) and it was constructed by using a lipid monolayer that contained DPPC: GM1 layer spontaneously was formed on the gold surface by exposing it to an aqueous dispersion of GM1-incorporated phospholipid vesicles. The quartz crystals could detect cholera toxin by mass changes on the sensing interface. The frequency responses of the developed piezoelectric biosensors were linearly related to the toxin concentration in the range of 0.25–1.0 colong mL^−1^ with a detection limit of 95 ng mL^−1^. This biosensor provides a practical rapid detector for cholera toxin.

A novel electrochemical aptasensor for the detection of Aflatoxin B1 (AFB1) based on glassy carbon electrodes modified with electropolymerized neutral red and polycarboxylated macrocyclic ligands in which DNA aptamers were covalently attached was reported in the literature [31]. The interaction with the toxin resulted in the decrease of the cathodic peak current that was measured by cyclic voltammetry (CV) and in the increase of the electron transfer resistance that was determined by electrochemical impedance techniques (EIS). The limit of detection was 1 nM for CV and 0.05 nM for EIS methods, respectively. The aptasensor was validated to the detection of AFB1 in peanuts, cashew nuts, white wine and soy sauce with a recovery of 85–100%.

A potentiometric nanosensor for cholera toxin has been proposed, using graphene nanosheets with incorporated lipid films [62]. GM1 was immobilized on the polymeric lipid films and provided good selectivity for the detection over a wide range of cholera toxin concentrations, rapid response times of ca. 5 min, and a detection limit of 1 nM. This device could be easily constructed and shown good reproducibility, reusability, selectivity, long shelf life and high sensitivity having a slope of 60 mV/decade of cholera toxin concentration. The method was evaluated and validated in lake water samples.

The state of art of the present paper are as follows: The biosensing surface can be easily and reproducibly be prepared and renewed, the lipid membrane interface at the biosensing surface provides a biocompatible environment that has proven beneficial in resisting nonspecific adsorption of anionic and cationic species, thus ensuring a low background signal in the assay. The proposed sensor reveals good reproducibility, reusability and selectivity along with high sensitivity fast response times (~5 min), nM detection limits, long shelf life, adequate operational stability for environmental analysis, it is constructed in a three-step, it is offered to be commercialized, it is without significant interference from other water constituents and can be used for routine analysis. The sensor has been successfully evaluated/ validated for cholera toxin detection in waters and the results show that the present GM1-cholera toxin interactions might serve as a suitable model for developing detection schemes for the detection of other receptor-mediated toxins such as *E. coli*, *Clostridium*, *Shigella* or *Pertussis* toxins and can be easily engineered into portable field detectors for onsite alarm monitoring

A miniaturized potentiometric saxitoxin sensor on graphene nanosheets with incorporated lipid films and anti-STX, the natural saxitoxin receptor has been recently described [15]. An adequate selectivity for detection over a wide range of toxin concentrations, fast response time of ca. 5–20 min, and detection limit of 1 nM have been achieved. The proposed sensor is easy to construct and exhibits good reproducibility, reusability, selectivity, long shelf life and high sensitivity of ca. 60 mV/decade of toxin concentration. The technique was implemented and evaluated in lake water and shellfish samples.

The sensor exhibits long shelf life and adequate operational stability for environmental and food analysis. The sensor has been validated for saxitoxin detection in waters and shellfish samples and the results show that the anti-STX-saxitoxin interactions employed herein might serve as a suitable model for developing detection schemes for other channel blocking toxins, such as brevetoxin, palytoxin or ciguatoxin. The state of art and advantages of this device are rapid response times (5–20 min, depending on toxin concentration) and nM detection levels without significant interference from other water constituents. The biosensing surface can be easily and reproducibly prepared and renewed. The lipid membrane interface at the biosensing surface provides a biocompatible environment that has proven beneficial in resisting nonspecific adsorption of anionic and cationic species, thus ensuring a low background signal in the assay. The proposed sensor reveals good reproducibility, reusability and selectivity along with a high sensitivity having a slope of ca. ~60 mV/decade over a wide logarithmic range of saxitoxin concentrations ranging from 1 × 10^−9^ M to 1 × 10^−6^ M. The proposed scheme can be easily packed into a portable field detector for rapid water quality preliminary assessment for life-threatening contaminants and bioterrorism attacks.

## 6. Conclusions and Future Trends

The present review paper provides methods of construction of biosensors based on lipid membranes and used for the rapid detection of a wide range of toxins (aflatoxin A1, M1 and B1, cholera toxin, etc.). These biosensors exhibit good selectivity, sensitivity and reproducibility, fast response times, they are easy to construct and can be used by non-skilled personnel for in the field measurements.

The release of toxins cause illness or death in people mainly, animals, or plants. Toxins can be found in nature or are prepared to cause diseases or death. Toxins are food toxicants or terrorism weapons used by terrorists as a method of creating mass panic and disruption to a society. Therefore, it is of primary concern to develop biosensors to detect toxins. Novel recent biosensor technology has significant technological advantages when compared to that of the traditional detection methods. However, biosensor technology still needs to construct a portable device for the rapid detection of Toxins that can be used for in the field measurements or in airports and other cases.

It is now possible to further explore the possibility of investigations of interactions of lipid membranes with other toxins. This novel ultrathin film technology can currently be adapted to the rapid detection of other toxins in foods and those that could be used in bioterrorism.
